# Indications, Techniques and Complications of Labiaplasty

**Published:** 2015-08-18

**Authors:** Arvind U. Gowda, Nikki Chopra, Marwan Khalifeh

**Affiliations:** ^a^Division of Plastic Surgery, University of Maryland School of Medicine, Baltimore; ^b^American University of Antigua School of Medicine, St George, Antigua; ^c^DC Cosmetics, Washington, DC

**Keywords:** labiaplasty, labiaplasty techniques, labial reduction, vaginal hypertrophy, vaginal rejuvenation

**Figure:**
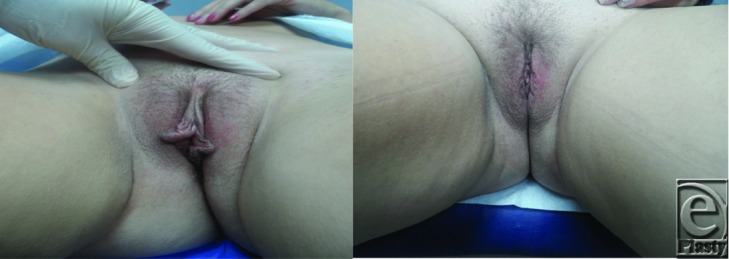


## DESCRIPTION

Labiaplasty is increasing in popularity. There is a lack of consensus regarding indications and techniques for this procedure. Despite an absence of accepted standards of practices, patient satisfaction rates are high. This article aims to provide a brief overview of labiaplasty.

## QUESTIONS

1. **What is vaginal labiaplasty?**
2. **What are the indications for labiaplasty?**
3. **What are the techniques for labia minora reduction?**
4. **What are the complications of labiaplasty?**

## DISCUSSION

Vaginal labiaplasty refers to surgical reduction of the labia minora. Additional goals of this procedure include minimal invasiveness, preservation of the introitus, optimal color/texture match, and maintenance of the neurovascular supply.^1^ Labiaplasty has become an increasingly popular in recent years.^1^

There are no widely accepted guidelines for labiaplasty, and it is carried out for a variety of reasons. Hypertrophy of the labia minora can cause dyspareunia, chronic urinary tract infections, irritation, hygienic difficulties, and interference with sports.^2^^,^^3^ What constitutes labial hypertrophy is poorly defined in the literature. Historically, authors have assigned varying distances from the midline to the lateral free edge of the labia minora as abnormal. Others have advocated for surgery only in the presence of chronic symptoms.^1^ More recently, a system taking into account labial protrusion as well as the distance from the lateral edge of the labia minora to that of the labia majora has been proposed.^1^ Although there is no established anatomic standard, reports suggest that woman prefer a prepubescent aesthetic, with the labia minora tucked within the confines of the labia majora.^1^ A study evaluating 131 patients undergoing labiaplasty found that 32% sought surgery for functional impairment or discomfort, 37% sought surgery for aesthetic purposes, and 31% sought surgery for a combination of these reasons.^3^ Because of poorly defined anatomic parameters and a lack of widely accepted indications, labiaplasty is somewhat controversial despite a high rate of patient satisfaction following the procedure.^4^

Several labiaplasty techniques have been described including deepithelialization, direct excision, wedge resection, and composite reduction. Deepithelialization removes a small amount of tissue while preserving the labial contour. It is best suited for patients with minimal hypertrophy.^1^ Direct excision is a straightforward approach to volume reduction; however, the aesthetic outcome is poor. The natural color, contour, and texture are lost. Furthermore, the scar may be visible.^5^ Wedge resection accomplishes a comparable volume reduction with direct excision while preserving the native labial contour.^1^ Composite reduction labiaplasty aims to correct clitoral protrusion and hooding in addition to labial reduction. Composite reduction is associated with a higher rate of complications and reoperation than other techniques.^6^ Additional procedures such as W-shaped resection, Z-plasty, and laser labiaplasty have been described in a small number of patients.^1^ Choice of technique should be based on patient anatomy, goals, and surgeon comfort.

The most common complications following labiaplasty are dehiscence, hematoma, unsatisfactory scarring, and superficial infections. In addition, flap necrosis has been reported with wedge resection.^1^

Labiaplasty is an increasingly popular procedure with high satisfactions rates, although the definition of labial hypertrophy and indications for surgery remain debated. Several techniques are available to accomplish labial reduction, and future studies are needed to establish practices optimizing patient care.
